# Isolated Lower Motor Neuron Facial Nerve Palsy in a Patient of Varicella: A Case Report

**DOI:** 10.31729/jnma.4849

**Published:** 2020-04-30

**Authors:** Sanjiv Choudhary, Ankita Srivastava, Soumya Narula

**Affiliations:** 1 Department of Dermatology, All India Institute of Medical Sciences, Nagpur, Maharashtra, India; 2 Department of Dermatology, All India Institute of Medical Sciences, Bhopal, Madhya Pradesh, India

**Keywords:** *corticosteroids*, *facial nerve palsy*, *treatment*, *varicella*

## Abstract

Varicella or chickenpox is usually regarded as a self-limiting disease, but occasionally it may lead to irreversible neurological complications. Isolated lower motor neuron facial nerve palsy is a rare complication of varicella, with very few cases reported from the Indian subcontinent. We report one such case, where the patient developed facial palsy after healing of cutaneous lesions and recovered completely with oral corticosteroids.

## INTRODUCTION

Varicella or chickenpox is a viral illness, caused by the varicella-zoster virus (VZV). It is a highly contagious disease that is commonly seen in childhood, but often adults are also affected. Although a self-limiting disease, occasionally serious systemic complications can occur in varicella, such as neurological, pulmonary, hepatic and renal involvement.^1^

Neurological sequelae as a result of primary VZV infection are rare and estimated to occur in approximately 0.01%-0.03% of cases.^2^ Most common of these neurologic complications are encephalitis, acute cerebellar ataxia, myelitis and meningitis.^3^ Here we report a case of isolated lower motor neuron facial nerve palsy in a case of varicella in an adult, which has been rarely described in the literature.

## CASE REPORT

A 22-years old male presented to the Out Patient Department (OPD) with complaint of deviation of angle of mouth to the right side since past 2 days. He gave a history of fever 12 days back that was followed by eruption of multiple, small, fluid-filled skin lesions all over the body. He took treatment from a nearby physician and was treated with antipyretics and antihistamines. No antiviral medication was prescribed to the patient. Fever subsided over the next 3-4 days and skin lesions also dried up in 1 week and he did not develop any new lesion and fever after that. Four days later, he suddenly developed deviation of angle of mouth towards the right side. There was no history of retro auricular pain, hyperacusis, and alteration in taste sensation. Cutaneous examination revealed multiple, discrete, 0.5-1 cm in size, brown to black crusted lesions over the face, trunk, and extremities.

**Figure 1.**
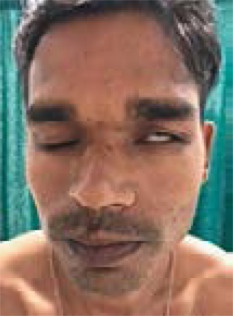
Left-sided facial nerve palsy with crusted skin lesions over the face.

The patient was unable to close the left eye with loss of forehead wrinkling on the left side (Figure 1).

Also, there was a loss of nasolabial fold on the left side. The deviation of the angle of the mouth towards the right side with the dribbling of saliva was noted. The rest of the neurological examination was within normal limits. An ophthalmological opinion was also sought, which revealed no abnormality. Computed tomography (CT) scan of the brain was normal. Serology for HIV-1 and HIV-2 virus was negative. Varicella-zoster virus (VZV) IgM antibody was found to be positive.

**Figure 2 (a,b).**
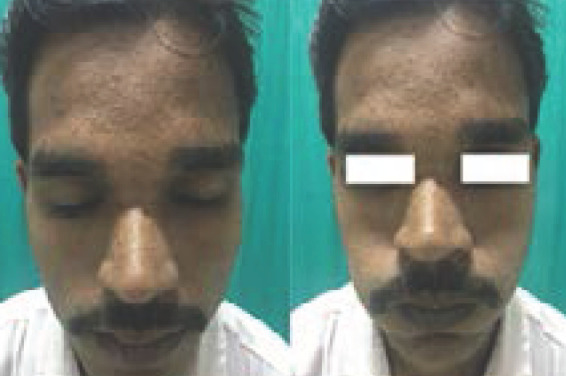
Complete recovery of facial nerve palsy after one month

On the basis of these findings, a diagnosis of varicella with isolated, left sided lower motor neuron facial nerve palsy was made and patient was started on oral corticosteroids (prednisolone at dose of 1mg/kg) and was tapered over 5 weeks and patient recovered completely after one month of corticosteroid therapy (Figure 2).

## DISCUSSION

Varicella-zoster virus (VZV) causes two distinct diseases-varicella or chickenpox and herpes zoster. Varicella occurs due to the primary infection that is common in childhood. Often, it is a self-limiting disease, but rarely it may result in severe neurological complications. Common CNS complications of varicella are cerebellar ataxia and encephalitis, and rare complications include transverse myelitis, aseptic meningitis, Guillain-Barre syndrome, meningoencephalitis, ventriculitis, optic neuritis, herpes zoster ophthalmicus, post-herpetic neuralgia, delayed contralateral hemiparesis, peripheral motor neuropathy, cerebral angiitis, Reye syndrome and facial paralysis.^4^ The various proposed mechanisms of these complications include direct neurological damage, immune-mediated damage and vasculopathy.^5^ Isolated facial nerve paralysis is a relatively rare complication of varicella.^6,7^ In a previous study, it was reported in 8.3% of the patients.^8^ The involvement may be unilateral or bilateral. The period between the appearance of the vesicles of varicella and the facial nerve palsy ranges from 5 days before and 16 days after the eruption.^9^ Therefore, the clinician needs to recognize that this neurological complication may develop even after subsidence of cutaneous lesions. This was seen in our case too, as the patient developed facial nerve palsy after 12 days of onset of fever and rash; when the fever had subsided and vesicular lesions had crusted. The proposed mechanisms of nerve damage in such cases include viral invasion of nerves or nerve damage due to immunologically mediated inflammatory responses.^6^

Other common causes of isolated facial palsy include Bell's palsy and Ramsay Hunt syndrome. Both of these conditions may be distinguished based on characteristic cutaneous features of varicella and can be confirmed by serological tests.

The prognosis of facial palsy due to varicella is generally good and 80% cases recover even without treatment. Though there are no well-established guidelines for treatment of varicella facial palsy, antivirals and corticosteroids are supposed to accelerate recovery.^10^ We treated our patient with corticosteroids alone as fever and eruption had already subsided. He showed an excellent response with complete recovery. Besides, adequate protection of eyes and physical rehabilitation is essential to prevent irreversible damage. Since the patient was not treated with antiviral drugs in the initial stage, this could have been a contributing factor for the development of neurological complications.

## Consent:

**JNMA Case Report Consent Form** was signed by the patient and the original article is attached with the patient's chart.
